# Pathways to diabetic care at hospitals in rural Eastern Uganda: a cross sectional study

**DOI:** 10.1186/s12913-019-3873-z

**Published:** 2019-01-14

**Authors:** Elizeus Rutebemberwa, James Bagonza, Raymond Tweheyo

**Affiliations:** 1 Department of Health Policy, Planning and Management, Makerere University School of Public Health, Kampala, Uganda; 2 African Centre for Health and Environmental Studies, Kampala, Uganda; 3 Migration Health Department, International Organization for Migration, Freetown, Sierra Leone; 4 Department of Public Health, Lira University, Lira, Uganda

**Keywords:** Pathways, Diabetes, Rural, Uganda

## Abstract

**Background:**

Prompt access to appropriate treatment reduces early onset of complications to chronic illnesses. Our objective was to document the health providers that patients with diabetes in rural areas seek treatment from before reaching hospitals.

**Methods:**

Patients attending diabetic clinics in two hospitals of Iganga and Bugiri in rural Eastern Uganda were asked the health providers they went to for treatment before they started attending the diabetic clinics at these hospitals. An exploratory sequential data analysis was used to evaluate the sequential pattern of the types of providers whom patients went to and how they transitioned from one type of provider to another.

**Results:**

Out of 496 patients assessed, 248 (50.0%) went first to hospitals, 104 (21.0%) to private clinics, 73 (14.7%) to health centres, 44 (8.9%) to drug shops and 27 (5.4%) to other types of providers like community health workers, neighbours and traditional healers. However, a total of 295 (59.5%) went to a second provider, 99 (20.0%) to a third, 32 (6.5%) to a fourth and 15 (3.0%) to a fifth before being enrolled in the hospitals’ diabetic clinics. Although community health workers, drug shops and household neighbours were utilized by 65 (13.1%) patients for treatment first, nobody went to these as a second provider. Instead patients went to hospitals, private clinics and health centres with very few patients going to herbalists. There is no clear pathway from one type of provider to another.

**Conclusions:**

Patients consult many types of providers before appropriate medical care is received. Communities need to be sensitized on seeking care early from hospitals. Health centres and private clinics need to be equipped to manage diabetes or at least diagnose it and refer patients to hospitals early enough since some patients go to these health centres first for treatment.

## Background

In 2016, the prevalence of Diabetes Mellitus from a national survey in Uganda was estimated at 1.4% and was higher in urban areas 2.6% compared to rural areas 1.0% [1]. However, another study that sampled a peri-urban and rural area in Uganda estimated the prevalence in the rural area at 16.1% and a peri-urban area at 7.6% [2]. A national survey in Tunisia showed an increase in diabetes in both urban and rural areas [3]. Unfortunately, the rural population has the highest proportion of the undiagnosed cases [4–6].

With the high burden of communicable diseases and a very low physician to patient ratio, primary health facilities in sub-Saharan Africa find it difficult to cope with the demanding care for non-communicable diseases [7]. In addition, there is low availability of medications for non-communicable diseases in low and middle income countries [8]. With an increasing prevalence of diabetes mellitus and low availability of appropriate medications, patients with diabetes could take long to reach the few hospitals that provide care on a regular basis.

In Uganda, some studies have explored health care seeking for diabetes. One study used semi-structured interviews and found that although care was sought from professional doctors and nurses, sometimes patients switched to alternative medicine due to perceived failure of modern medicine [9]. Another study which used focus group discussions also highlighted that patients routinely switch their healthcare between different providers [10]. Within the Ugandan setting, self-referral between primary care providers has been documented [11]. The person or facility they go to first after deciding to get treatment outside the home was considered as the first provider. The second provider was the person or facility they would go to after the first one. The third provider was the one they would go to after the second one and so forth. The objective of this study was to describe how patients with diabetes switch among different providers before they get treatment from hospital-based diabetic clinics.

## Methods

### Study area

The study was conducted in two public hospitals–Iganga and Bugiri. The two hospitals are the main treatment centres for diabetes within the districts of Iganga and Bugiri. They act as referral centres for the surrounding lower level facilities, private clinics and drug shops. As with other public hospitals in Uganda, patients can however access them directly without passing through any lower level health facility.

When people get sick in Iganga and Bugiri districts, they have different types of providers to go to; hospitals, health centres, private clinics, drug shops, Community Health Workers (CHWs), herbalists, neighbours or friends, or others like preachers and pastors. Neighbours could be close relatives like in-laws as has been reported in some studies [12] or a close friend [13]. Health centres are government owned health facilities that are lower than the hospital. They operate mainly outpatient services although a few may offer inpatient care. There are three levels of health centres in Uganda where health care could be sought below the hospital namely; Health Centre II, Health Centre III and Health Centre IV. Health Centre II provides outpatient care and community outreaches, Health Centre III adds on basic laboratory diagnosis and maternity care. Health Centre IV carries out minor operations in addition to what the lower level health centres do [14]. Health centres in Iganga and Bugiri districts have for long been suffering from low staffing levels and inadequate funding like many other facilities in Uganda [15].

Private clinics are small scale health facilities that operate mainly on outpatient basis. Some of the clinics have basic laboratory services like microscopy. Some are owned or registered under a formal health provider’s name, who may be in full time private practice or works in another government facility. These clinics are managed on a day-to-day basis either by the owner supported by other health workers, or in most cases, by a nurse, midwife or nursing aide [16].

Drug shops mainly sell medicines to patients. They may do so on prescription or not. Some drug shops provide clinical management depending on who is attending to patients at the time although others don’t [16]. When the person attending to the patients is a trained health worker like a nurse or clinical officer, that person may first ask the patient questions and come up with a tentative diagnosis that will guide the type of drugs to give the patient. However, when the person left in the drug shop is not trained in a medical field, that person would just give whatever the patient has requested for even without a prescription. Many of these drug shops are unregistered and their quality of service is sub-optimal [17, 18].

Community health workers are members of the communities where they work, are selected by those communities, usually to support a health care program like the integrated community case management of childhood illnesses [19]. They may also be used to support other programs like community based directly-observed short course treatment for tuberculosis. CHWs do not get regular payment for their services. However, individual CHWs are often utilized by different health programs in community interventions, which increases their social standing and prestige [20]. They carry out most of their mandated tasks from their homes.

Herbalists are a heterogenous group. Some use only traditional medicine in their practice while others mix western and traditional medicines [21]. Traditional medicine is mainly medicinal plants but others could be incantations or prayers [22]. Herbalists also operate mainly from their homes or in markets on market days [23].

#### Study population and data collection

Study participants were patients with diabetes mellitus (either Type 1 or Type 2) attending diabetic clinics in Iganga and Bugiri hospitals. Diabetic patients who sought treatment from these two hospitals on diabetic clinic days from October 2012 to March 2013 were included in the study. Exclusion criteria were having diabetic complications or coming to the hospital on a day that was not put aside for the diabetic clinic. Data collection was by use of a questionnaire, and data was cross-checked with medical records where applicable. Data was collected only on diabetic clinic days when patients came for checking blood sugar levels and for drug refills.

Patients were asked from where the first treatment was received, for the symptoms that brought them to the hospital. If the first treatment provider was not the current study hospital, they were then asked where they went after their primary treatment place. If the current provider was not the place where they were receiving treatment from, they were again asked where they went after that. The same questioning routine was continued to exhaust all the previous treatment providers prior to the current study site/ hospital. The specific questions that were asked were: What treatment did you give at home immediately you realized that you had a problem before seeking treatment elsewhere? When you realized you were not well and needed medical attention, where did you go to seek treatment first outside the home? Where did you go after the first place? Why did you shift from the first place? Where did you go after the second place? Why did you shift from the second place? These questions were continued until when the facility indicated was the hospital where the interview was taking place.

Data was collected by nurses and clinical officers working in the diabetic clinics of these hospitals. Nurses and clinical officers working in the diabetes clinics of the study sites were trained for data collection. They were selected because they routinely assess and evaluate all the diabetic patients who come to the hospital. This was not expected to bring any bias to the results, since what was asked in the study is the same as what these health workers ask routinely in history taking. Study information was collected after explaining to the patients the purposes of the study. The interviews were conducted in a room where the diabetic clinic takes place. This consideration enabled privacy, and offered the patients a conducive atmosphere to discuss diabetes since the general population sometimes fears patients with diabetes [12].

#### Data management and analysis

Filled questionnaires were checked for completeness on a daily basis, then collected from the diabetes clinic every fortnight and taken to a central place for data entry. Data entry and analysis was done in Epi-info version 3.5.1. Data analysis used principles of exploratory sequential data analysis to evaluate the pathways of diabetic care. The analysis was based on the method used by Ryan (1998) for the analysis of sequential data of healthcare seeking for lay people in rural Cameroon [24]. The sequential pathways analysis has previously been validated in studies exploring pathways to psychiatric care in Eastern Europe [25], and tuberculosis care in China [26], and for medical pluralism among HIV/AIDS patients in South Africa [27]. Principally, a sequential analysis is used to systematically evaluate sequential events, for depicting the chronological occurrence of distinct events, thus examining underlying behavioral patterns [24, 25, 28]. We had three units of analysis, (i) “the location of the care-provider” for a diabetic patient, such as a CHW, private clinic or hospital, (ii) “the ordered choice of care-provider for each diabetic patient”, for example first, second, third and other, and (iii) “transitioning between providers”.

**Fig. 1:**
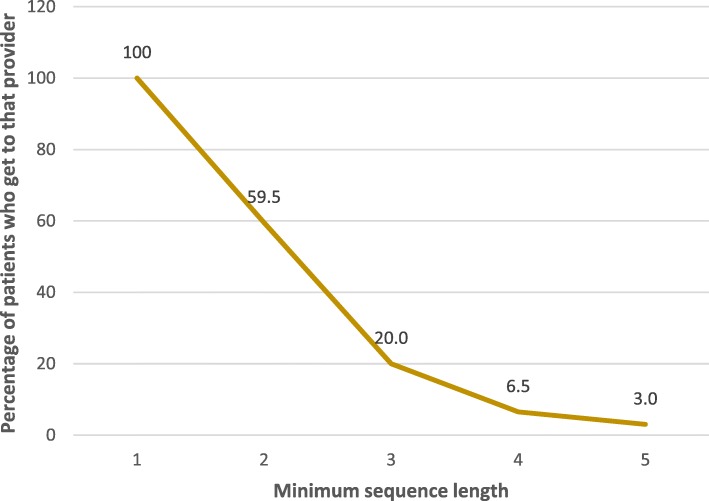
Scree plot showing number of providers seen (minimum sequence length) by the participants (*n* = 496)

**Fig. 2:**
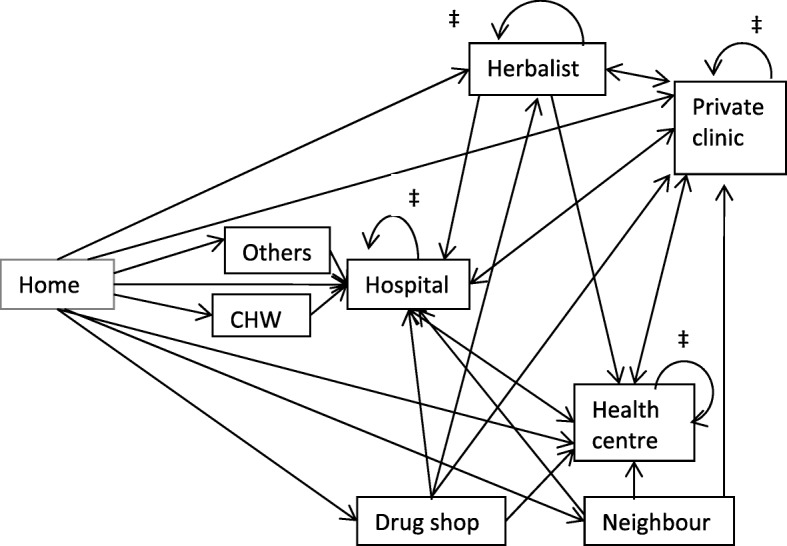
Transitions from one provider to another (*n* = 496). ‡The curved arrows indicate that a patient moved from one provider to another provider in that category

Firstly, the analysis answered the question, ‘what are the characteristics of the participants?’ in terms of sex, marital status, education completed, occupation, age and time spent having diabetes. This is given in Table 1. Secondly, it was important to determine ‘what proportion of patients visited a first provider, second, third, fourth or fifth prior to getting care at the hospital?’ The sequence length for the analysis was limited to the first five consultations. Specifically, data was analysed by tabulating proportion of participants who went for the first time, second, third, fourth or fifth time. This is presented in a scree plot (Fig. 1) depicting the decreasing proportion of participants as the number of providers seen increased. Thirdly, the analysis explored the question, ‘what was the patient’s sequence of care seeking?’ For this, a descriptive care pathway was developed (Fig. 2). There was potential for multiple combinations of care-seeking pathways, with patients transitioning between various providers. For example, a patient could go to a drug shop, followed by a private clinic, then finally to a hospital, while another could first visit a hospital, then a private clinic, followed by a health facility, and back to a hospital. Some went to the hospital the first time and stayed there. Others would reach the hospital as a second provider and stay there and so on. To assess for a possibility of memory loss in those who had been on treatment for a long time, a sub-analysis of those who had been with diabetes for not more than 1 year was done. Fourthly, at each transition from a provider to the next, like provider one to provider two and the subsequent providers seen, participants were asked why they switched providers. This is shown in Table 2. The denominator is the number of people who made that transition. A bivariate analysis was done on the association between having formal education and going to the hospital as a first provider. Next, the analysis answered the question, ‘how frequently are care providers used?’ Similar to previous studies [24, 25], proportions were computed for care seeking – at each service provider before reaching the current provider (hospital) – Table 3.

**Table 1:** Participants’ characteristics

Variable	Frequency (*n* = 496)	Percentage
Sex		
Female	251	50.6%
Male	245	49.4%
Marital status		
Married / cohabiting	367	74.0%
Separated / divorced	35	7.1%
Single / never married	27	5.4%
Widowed	67	13.5%
Education completed		
None	229	46.2%
Primary	171	34.5%
Secondary S4	57	11.5%
Secondary S6	10	2.0%
Tertiary	29	5.8%
Occupation		
Artisan	11	2.2%
Farming	271	54.6%
Government employee	43	8.7%
Housewife	24	4.8%
Retired worker	13	2.6%
Trader	90	18.1%
Unemployed	44	8.9%
Agegroup		
< =20	15	3.0%
> 20–30	25	5.0%
> 30–40	82	16.5%
> 40–50	149	30.0%
> 50–60	124	25.0%
> 60–70	69	13.9%
> 70	32	6.5%
Time diabetic (months)		
< =12	108	21.8%
> 12–24	61	12.3%
> 24–36	66	13.3%
> 36–48	35	7.1%
> 48–60	50	10.1%
> 60–72	40	8.1%
> 72–84	25	5.0%
> 84–96	26	5.2%
> 96	85	17.1%

**Table 2:** Reasons for shifting from one provider to another

Reason for moving to next provider	From first to second (*n* = 295)	From second to third (*n* = 99)	From third to fourth (*n* = 32)	From fourth to fifth (*n* = 15)
1. Better care and treatment	203 (68.8%)	62 (62.6%)	15 (46.9%)	9 (60.0%)
2. High transport costs	60 (20.3%)	28 (28.3%)	6 (18.8%)	2 (13.3%)
3. Treatment being expensive	20 (6.8%)	3 (3.0%)	1 (3.1%)	0 (0.0%)
4. Others^a^	12 (4.1%)	13 (13.1%)	10 (31.3%)	4 (26.7%)

^a^ Others included personal reasons like change of job or being near relatives

**Table 3:** Number of times health providers were chosen seeking treatment for diabetic symptoms

Provider		First place *N* = 496 (%)	Second place *N* = 295 (%)	Third place *N* = 99 (%)	Fourth place *N* = 32 (%)	Fifth place *N* = 15 (%)
Hospital	Total	248 (50.0)	224 (75.9)	78 (78.8)	24 (75.0)	13 (86.7)
	Female	122 (48.6)	124 (76.5)	48 (82.8)	13 (72.2)	8 (100)
	Male	126 (51.4)	100 (75.2)	30 (73.2)	11 (78.6)	5 (71.4)
Health Centre	Total	73 (14.7)	27 (9.2)	10 (10.1)	5 (15.6)	2 (13.3)
	Female	32 (12.7)	12 (7.4)	5 (8.6)	3 (16.7)	0 (0.0)
	Male	41 (16.7)	15 (11.3)	5 (12.3)	2 14.3)	2 (28.6)
Private clinic	Total	104 (21.0)	40 (13.6)	10 (10.1)	3 (9.4)	0 (0.0)
	Female	55 (21.9)	25 (15.4)	5 (8.6)	2 (11.1)	0 (0.0)
	Male	49 (20.0)	15 (11.3)	5 (12.3)	1 (7.1)	0 (0.0)
Herbalist	Total	6 (1.2)	4 (1.4)	1 (1.0)	0 (0.0)	0 (0.0)
	Female	3 (1.2)	1 (0.6)	0 (0.0)	0 (0.0)	0 (0.0)
	Male	3 (1.2)	3 (2.3)	1 (2.4)	0 (0.0)	0 (0.0)
Drug shop	Total	44 (8.9)	0 (0.0)	0 (0.0)	0 (0.0)	0 (0.0)
	Female	22 (8.8)	0 (0.0)	0 (0.0)	0 (0.0)	0 (0.0)
	Male	22 (9.8)	0 (0.0)	0 (0.0)	0 (0.0)	0 (0.0)
Neighbour	Total	15 (3.0)	0 (0.0)	0 (0.0)	0 (0.0)	0 (0.0)
	Female	13 (5.2)	0 (0.0)	0 (0.0)	0 (0.0)	0 (0.0)
	Male	2 (0.8)	0 (0.0)	0 (0.0)	0 (0.0)	0 (0.0)
CHW	Total	3 (0.6)	0 (0.0)	0 (0.0)	0 (0.0)	0 (0.0)
	Female	3 (1.3)	0 (0.0)	0 (0.0)	0 (0.0)	0 (0.0)
	Male	0 (0.0)	0 (0.0)	0 (0.0)	0 (0.0)	0 (0.0)
Other	Total	3 (0.6)	0 (0.0)	0 (0.0)	0 (0.0)	0 (0.0)
	Female	1 (0.4)	0 (0.0)	0 (0.0)	0 (0.0)	0 (0.0)
	Male	2 (0.8)	0 (0.0)	0 (0.0)	0 (0.0)	0 (0.0)

**Fig. 3:**
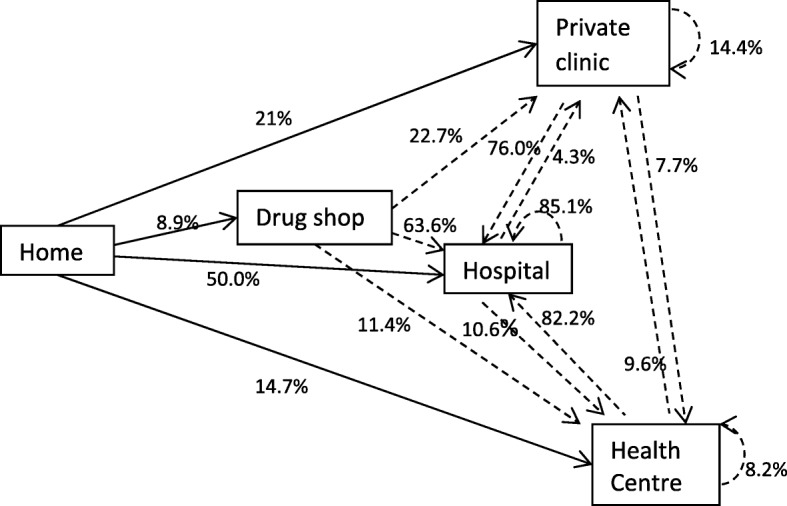
Care pathway showing the movement of participants to their first (continuous line) and second providers (dotted lines)

We conducted further analysis for the significance of the first two pathways between providers, considering as significant the transitions involving more than 5% of participants [24, 25, 28] thus leading to the development of a care pathway diagram showing transitions between home and the two most important providers (Fig. 3). The four providers that took more than 5% of the participants from their homes are indicated with continuous arrows. The denominator is the total number of participants in the study as all of them visited at least one care provider. The movement from the first provider to second provider with more than 5% of the participants from the first provider is indicated with a dotted line. The percentages on the dotted lines represent the proportion moving from provider say ‘x’ to provider ‘y’ as a percentage of all the participants that used provider ‘x’ as first provider and moved to any second provider.

## Results

### Characteristics of the participants

A total of 496 patients were interviewed from the two hospitals with Iganga contributing 278 (56%) and Bugiri 218 (44%). Females were 251 (50.6%), participants cohabiting or married were 367 (74%). A total of 229 (46.2%) patients did not have any formal education, (271) 54.6% were engaged in farming and 273 (55%) were aged between 40 and 60 years (mean 50.2 years and SD 14.2 years) (Table 1). Participants were asked the time they had spent on treatment for diabetes. A total of 85 (17.1%) patients had been on treatment for more than 8 years although those who had just started treatment for a year or less were 108 (21.8%). The time spent on treatment was confirmed from their medical records in files at the diabetes clinic.

### Providers seen before coming to hospitals

The index primary care provider for most of the participants was a hospital, 248 (50.0%), followed by private clinics 104 (21.0%), health centres 73 (14.7%), drug shops 44 (8.9%), and others such as CHWs, household neighbours or even traditional healers. Participants who went to a second provider were 295 (59.5%), while those who went to a third provider were 99 (20%), to a fourth provider 32 (6.5%), and a fifth provider 15 (3.0%). Figure 1 shows the proportion of patients who went to one, two, three, four or five providers before reaching the hospital where they were receiving treatment. Patients shift from one provider to another for various reasons. The majority said they shifted from first provider to the second provider because they were seeking better care and treatment 203/295 (68.8%) as shown in Table 2. High transport costs 60/295 (20.3%) and seeking less expensive treatment 20/295 (6.8%) were the other two major reasons.

The relationship between the education status of the patients and whether they sought treatment from the hospital first was assessed. Out of the 229 patients who had no formal education, 107/229 (46.7%) reported seeking care from hospitals as first provider. Of those who had received formal education, 141/267 (52.8%) sought care from hospital as first provider. There was no significant association between having formal education and having sought care from hospital as first provider (OR = 0.78; 95% CI 0.55–1.12, *p*-value 0.089).

### Transitions from one provider to another

After the first health provider, only four types of providers were selected as a second provider namely: hospital, private clinic, health centre or herbalist. This is illustrated in Fig. 2. To be noted is that 6/73 (8.2%) of those who had gone to health centres as first provider still went to health centres as their second provider, 1/6 (16.7%) of those who had gone to a herbalist as first provider went to another herbalist as second provider, 40/47 of those who had gone to a hospital as first provider went to another hospital as second provider and 15/104 (14.4%) who had gone to a private clinic went to another private clinic as second provider. However, all those who went to CHWs or other providers like preachers and pastors went to the hospital as their second place of choice.

In order to measure the significance of a pathway [24], an analysis was done for the provider who received at least 5% of the participants from the first provider where care was sought. Only drug shops, private clinics, hospitals and health centres saw more than 5% of the patients from their homes. Drug shops saw 44 patients and of these, 5/44 (11.4%) went to health centres, 28/44 (63.6%) went to the hospital, 10/44 (22.7%) went to a private clinic and 1/44 (2.3%) went to an herbalist as second provider. Private clinics saw 104 patients as first provider and of those, 79/104 (76.0%) went to hospital, 15/104 (14.4%) went to other private clinics, 8/104 (7.7%) went to health centres and 2/104 (1.9%) went to an herbalist as second provider. Of the 248 who went to hospital as first place for treatment, 47 of these moved to another facility with 40/47 (85.1%) going to another hospital, 5/47 (10.6%) going to health centres and 2/47 (4.3%) went to a private clinic as second place for treatment. Of the 73 that had gone to health centres, 60/73 (82.2%) went to hospitals as second place of call, 7/73 (9.6%) went to private clinics and 6/73 (8.2%) went to other health centres. This is demonstrated in Fig. 3.

### Sequence in which health providers were seen

A total of 248/496 (50%) of the patients with diabetes at these two hospitals had the hospital as their first place of call, while for those who went to a second provider, 224/295 (75.9%) went to hospitals while for the third, fourth and fifth provider, it was 78/99 (78.8%), 24/32 (75.0%), and 13/15 (86.7%) respectively. Stratification was done between males and females. This is illustrated in Table 3.

Since 85/496 (17.1%) patients had been on treatment for more than 8 years, there could have been recall bias on the providers they saw before coming for treatment at these diabetic clinics. An assessment was therefore conducted on only those who had just started treatment within 1 year or less. Proportions of those who went to the different providers as their first provider among those of a year or less (108 patients) were compared with proportions among all the 496 patients. Among the 108 patients who had been on treatment within a year or less, those who went to hospital first were 48/108 (48.8%) compared to 248/496 (50%) of all respondents. For health centres they were 14/108 (13.0%) compared to 73/496 (14.7%), those who went to private clinics were 18/108 (16.7%) compared to 104/496 (21.0%), and those who went to drug shops were 13/108 (12.0%) compared to 44/496 (8.9%). The figures were not very different among four major health providers – the hospitals, health centres, private clinics and drug shops.

## Discussion

This study highlights four issues in the way patients with diabetes seek care from different types of providers before reaching specialist diabetic clinics at hospital level. 1) A total of 15/496 (3.0%) saw up to five providers before they got to the hospital. 2) Half of the patients who noticed symptoms of diabetes went to hospital as their first place of call and even for those who went to second, third, fourth or fifth provider, they eventually ended up in hospitals. However, this could be due to getting our study participants from hospitals. 3) Hospitals, health facilities and private clinics attend to a significant number of patients with diabetes. 4) Herbalists were some of the providers that patients would keep going to as the second or third provider.

Our study shows that some patients with diabetes consult many providers before they reach the hospital diabetic clinic that provides them with treatment on a regular basis. The reasons they gave for shifting from one provider to the next were mainly to seek for better treatment although transport and expenses were also mentioned. Patients consult various providers other than the hospital before getting to a definite diagnosis. Switching providers for diabetes has been highlighted in other previous studies [9, 10]. A previous study in Iganga district indicated that some patients with diabetes first think of it as HIV or witchcraft [12]. Patients often first seek care from health facilities closest to their homes, irrespective of their illness. They shift from one provider to another due to costs, proximity or quality of care [29]. An important health system challenge is that high level facilities that can diagnose diabetes in rural areas are not easily accessible and this leads to delay to diagnose and appropriately treat diabetes. There is usually lack of expertise to manage diabetes at primary health care levels [30]. For example, research in neighbouring Tanzania shows many of their lower facilities lack diagnostic equipment, may not be having guidelines and lack anti-diabetic medicines [31, 32]. Switching between multiple providers also attests to limited capacity for the continuity of care for diabetes, a weakness reported for the management of chronic illnesses in sub-Saharan Africa [7].

Results from our study also indicate that in the long run, patients get care from the public hospitals. Previously, some studies conducted in Uganda indicated that most of the ambulatory care is provided by the private providers who are more numerous and spread out than the government facilities. However, long term care is mostly provided from the public facilities [29]. Diabetes is a chronic illness and hospitals are the main providers for this care even in rural areas. Unfortunately, several rural public hospitals often run short of medicines and this leaves the patients with fewer alternatives, with some opting for traditional medicine [23]. It is therefore critical for government to adequately finance hospitals to manage chronic illnesses like diabetes, because hospitals are ultimately the places from which patients with diabetes seek care.

Hospitals, health centres and private clinics are providers that receive significant numbers of patients who move from their initial provider. Drug shops are also one of the options that patients first seek treatment from although all these patients shifted to a different second provider. The frequent movement of patients between providers highlights challenges of referral within the health care system, but also that some providers are transit points as patients move to higher levels of care. Health centres and private clinics still receive patients as third place of call despite these facilities not having adequate capacity to handle patients with diabetes. Hospitals are few and far apart. It is critical therefore that drug shop attendants be equipped with skills to refer the diabetic patients promptly. Providers in health centres and private clinics should be trained to handle patients with diabetes since patients go to them even after receiving treatment from their first provider. If diabetes care services are to be brought near to the people, at the minimum, capacity needs to be built in lower-level health facilities, so that diabetic patients who often return to them for care access timely and appropriate treatment.

Patients with diabetes continue to seek care from herbalists even as second or third provider. Since data was collected through self-reporting, it is possible that this is an underestimation. Such a practice was already identified even among those who were attending hospital clinics for their treatment [23]. This calls for community sensitizations to increase the understanding of the general population on diabetes, a need for system strengthening to ensure constant supply of medicines and availability of personnel at health facilities and policy-level discussions on whether diabetic treatment could be provided at lower facilities to reduce on the transport costs of the patients.

There was no significant difference between those with formal and those without formal education in choosing hospitals as their first health provider when they noticed diabetic symptoms (OR = 0.78; 95% CI 0.55–1.12). This is in contrast with other studies that highlighted low formal education as a barrier to diabetes treatment [33, 34]. It is important to note that almost half of study participants had never had any formal education and less than a quarter of the respondents had an education level beyond primary education. Further studies could explore why those who have had formal education do not significantly differ from those who have never had formal education with respect to choosing hospitals as their first provider.

### Methodological considerations

The first limitation is that some patients had been on treatment for a long time, with 85 (17.1%) having been on treatment beyond 8 years. This could have affected their capacity to recall events leading to their enrolment into treatment at the diabetic clinics. However, when we considered only those who were on treatment for 1 year, the proportions that went to the respective providers were similar. We also asked them significant events that could be remembered over a long time and since diabetic patients are reviewed frequently, the way they had sought care should keep coming back to their minds. In addition, the health providers that one seeks care from is part of the routine history taking in a health care facility. It is therefore more likely that these patients had over some time been giving that history as they sought treatment. The second limitation is that there could have been response bias where patients may give desired answers since the interviewers were health workers. For example, patients who went to herbalists may be under estimated since it is not desirable to tell health workers that one sought care from herbalists. The third limitation was that the study asked the sequence of seeking care from health providers but sometimes, patients seek care from two or more providers concurrently like from hospitals and the traditional healers especially when patients take both modern and traditional medicine for the same illness. This was difficult to mitigate. However, looking at the sequence that patients mentioned in going to specific providers could give a picture of the number of providers where patients with diabetes seek treatment during the course of their illness. In this study, we restricted ourselves to the pathway up to their first contact with the hospital. It is also possible that even when patients are receiving treatment from a diabetic clinic, sometimes, they may go to these other providers when there are no medicines at the clinics, staff absenteeism, or inability to meet transport costs [35]. Sometimes hospitals in Uganda lack medicines and diagnostic equipment for diabetes [36]. Those patients with diabetes who were seeking treatment from other areas other than hospitals were missed. They would certainly depict a different pathway and another study done with different providers as end points may demonstrate a more comprehensive picture of pathways of patients with diabetes.

## Conclusion and recommendations

Patients with diabetes consult many providers before reaching hospitals. Proper education needs to be in place to strengthen patients’ knowledge. Health centres and private clinics need to be equipped with capacity to treat patients with diabetes because patients keep going there for treatment even after moving from their first provider. Diabetes care services in hospitals need to be well financed and resourced with skilled personnel, medicines, equipment and supplies, because a majority of patients with diabetes access care initiation and follow-up services from them.

## Abbreviation

CHW: Community Health Worker
